# The role of DNA-binding and ARNT dimerization on the nucleo-cytoplasmic translocation of the aryl hydrocarbon receptor

**DOI:** 10.1038/s41598-021-97507-w

**Published:** 2021-09-14

**Authors:** Rashad Haidar, Frank Henkler, Josephine Kugler, Aline Rosin, Doris Genkinger, Peter Laux, Andreas Luch

**Affiliations:** 1 Department of Chemical and Product Safety, German Federal Institute for Risk Assessment (BfR), Berlin, Germany; 2 Department of Biology, Chemistry and Pharmacy, Institute of Pharmacy, Freie Universität Berlin, Berlin, Germany

**Keywords:** Biochemistry, Biotechnology, Cell biology, Molecular biology

## Abstract

The human aryl hydrocarbon receptor (AHR) is predominantly located in the cytoplasm, while activation depends on its nuclear translocation. Binding to endogenous or xenobiotic ligands terminates the basal nucleo-cytoplasmic shuttling and stabilizes an exclusive nuclear population. The precise mechanisms that facilitate such stable nuclear accumulation remain to be clarified as essential step in the activation cascade. In this study, we have tested whether the sustained nuclear compartmentalization of ligand-bound or basal AHR might further require heterodimerization with the AHR-nuclear translocator (ARNT) and binding to the cognate XRE-motif. Mutagenesis of the DNA-binding motif or of selected individual residues in the ARNT-binding motif did not lead to any variation in AHR’s nucleo-cytoplasmic distribution*.* In response to ligands, all mutants were retained in the nucleus demonstrating that the stable compartmentalization of activated AHR in the nucleus is neither dependent on interactions with DNA, nor ARNT. Knocking down the ARNT gene using small interfering RNA confirmed that ARNT does not play any role in the intracellular trafficking of AHR.

## Introduction

The aryl hydrocarbon receptor (AHR) is a ligand induced transcription factor that activates oxidative phase I metabolism by the induction of cytochrome P450-dependent monooxygenases (*CYP*), especially *CYP1A1* and *1B1*^1^. Early-identified AHR ligands included environmental toxicants, as for example 2,3,7,8-tetrachlordibenzo-*p*-dioxin (TCDD)^2^ and benzo[*a*]pyrene (B[*a*]p)^3^, as well as β-naphthoflavone (BNF)^4^ and other xenobiotic compounds, thereby pointing to the role of the AHR in xenobiotic substance metabolism. However, more current studies demonstrate the AHR to be more than a simple sensor, indicating further functional interactions with other signalling pathways^5,6^. The AHR plays also a major role in organ development^7,8^, regulation of adaptive immunity^9^ and the maintenance of lung health^10^. Furthermore, several endogenous ligands have been identified consistent with various physiological functions, such as skin differentiation^11^ or intestinal homeostasis^12,13^. Endogenous AHR agonists include tryptophan metabolites, such as kynurenine (KYN), and indole derivatives, such as indirubin (IND)^14^. The latter is confirmed to be present in the urine of healthy people with levels sufficient to activate the AHR^15^. Recent research has shown that bacterial products from *Pseudomonas aeruginosa* can bind and activate the AHR, which indicates that the AHR collaborates in antibacterial responses^16^.

Activation of the AHR is linked to increased oxidative metabolism and consequently the formation of, e.g., reactive oxygen species. In fact, prolonged activation of the AHR can enhance carcinogenic effects^17^. Transcriptional activation of the AHR is therefore a tightly regulated process that depends on the interactions with several co-factors and the heterodimerization with the AHR-nuclear translocator (ARNT). ARNT is a nuclear protein that acts as dimerization partner for several transcription factors including hypoxia-inducible factors (HIF)^18^, single-minded proteins (SIM)^19^ or the estrogen receptor (ER)^20^. Studies on the anti-estrogenic effects of AHR-activation confirmed the competition between AHR and ER to interact with ARNT^21^.

The highly conserved bHLH domain within the N-terminal domain contains a bipartite nuclear localisation signal (NLS), adjacent to a nuclear export signal (NES). These highly conserved motifs regulate the intracellular trafficking of the AHR. Notably, the DNA-binding motif of the AHR overlaps with the NLS, more accurately with the amino acids Histidine 39 (H39) and Arginine 40 (R40) in the second NLS^22–24^.

Activation of the AHR is strongly linked to intracellular transport. Prior to binding to inducing ligands, the AHR is mainly located in the cytoplasm^25^ as a part of a chaperon complex consisting of HSP90, XAP2 and p23^26^. The HSP90 masks the NLS to prevent nuclear import^26^. The current model of the AHR signalling pathway describes ligand-binding as key event that triggers a conformational change^27^, which unmasks the NLS and exposes it to importin-α. The latter binds to importin-β to pass the nuclear protein import^28^. However, continuous shuttling of the AHR between cytoplasm and nucleus is confirmed in the absence of ligands^23,29^. In the context of shuttling, basal import is efficiently compensated by NES-dependent export that maintains a pre-dominantly cytoplasmic compartmentalization. Notably, this shuttling does not lead to any activation^29^ and its import related-mechanism, with regard to the NLS, is still not well understood.

During activation, AHR is released from its chaperon complex in the nucleus and forms a heterodimer with ARNT^30^. The AHR-ARNT dimer binds to a cognate sequence in the promoter region of AHR target genes, the xenobiotic response elements (XRE)^31^. This binding is accelerated by numerous coactivators that associate with the AHR-ARNT dimer to enhance the promoter accessibility^32^. In order to initiate transcription, receptor-ligand complexes need to engage with several co-factors and DNA, involving both the N- and C-terminal domains. Importantly, this needs to occur within the limited time interval of nuclear transit, since the export of the AHR from the nucleus continues in the presence of ligands^29^. The nuclear mechanisms involved in terminating export and subsequently stabilizing the nuclear population are important steps towards activation. However, these steps are not yet fully understood.

In principle, associations with ARNT as well as binding to the DNA and other nuclear factors might anchor the AHR in the nucleus, thus facilitating further steps towards transcription. However, this theory has not yet been tested. As alternative option, ligand-binding might inactivate export into the cytoplasm, possibly due to conformational changes that masks the NES. This could terminate endogenous shuttling, leading to a constitutive nuclear population to engage in various functional interactions. The NES is also postulated to terminate transcription by re-exporting the receptor into the cytoplasm^29^, where it can be degraded by the proteasome^33^.

In this study, we have investigated whether the ligand-induced nuclear accumulation of the AHR depends on its heterodimerization with ARNT and its DNA-binding domain that associates with the cognate XRE motif. Our data indicate the nuclear compartmentalization is not affected or stabilized by consecutive association with either DNA or ARNT. Instead, the alternative concept of an inhibited NES-dependent export during receptor activation is supported.

## Results

### Using the AHR crystal structure to generate AHR-mutants that are deficient for DNA-binding and/or dimerization with ARNT

To design AHR mutants that are incapable of interacting with ARNT or the XRE, we took reference to the AHR crystal structure model as defined by^34^*.* The quoted crystal structure refers to an AHR-ARNT complex, containing only the bHLH and PAS-A domains of both proteins, bound to a 12mer double-stranded DNA. Crucial residues that are involved in both interactions with ARNT or the XRE have been identified or confirmed in agreement with previous functional studies^35,36^.

**Figure 1:**
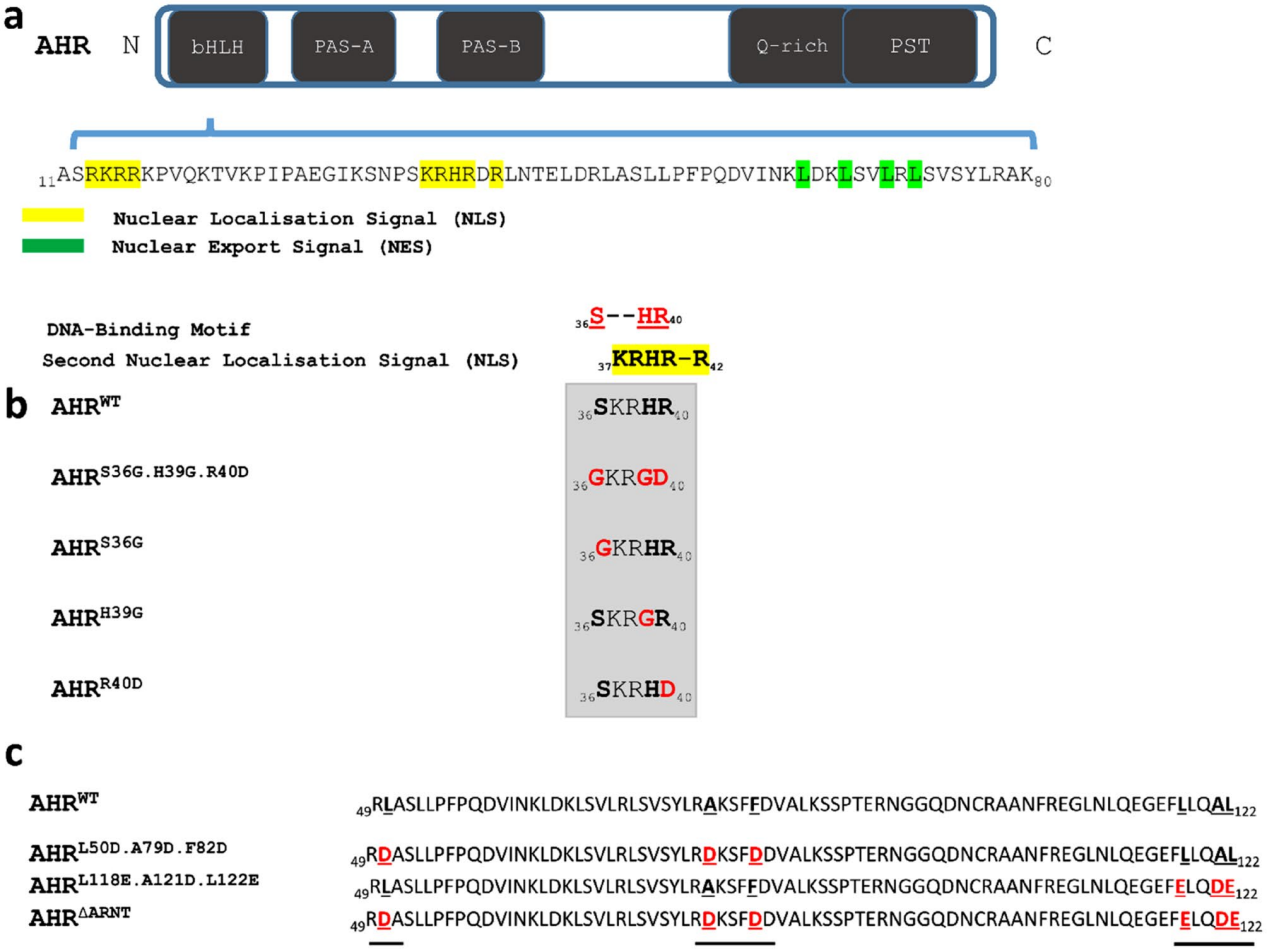
Design of AHR mutants to study DNA-binding and ARNT dimerization. (**a**) Domain architecture of the AHR. The bipartite nuclear localization signal (NLS) and a nuclear export signal (NES) are indicated as amino acid sequence. Graphical representation of mutated amino acids in the DNA-binding motif (**b**) and ARNT-binding domain (**c**) of the AHR.

According to the crystal structure, the interaction of AHR with the XRE motif relies on three amino acids: Ser36 (S36), His39 (H39) and Arg40 (R40). While S36 and R40 form hydrogen bonds with cytosine and guanine on the DNA strand, H39 builds a contact to the phosphate backbone of the thymine base^34^. For an entire characterization profile of the DNA-binding motif, we examined different AHR-mutants covering the whole DNA-binding motif as well as the individually mutated amino acids (Fig. 1b).

The dimerization of AHR and ARNT includes three interaction interfaces in the bHLH as well as the PAS-A domain. In accordance with this information the amino acids L50, A79, F82, L118, A121, L122 have been identified as crucial for the formation of a stable AHR-ARNT heterodimer^34,36^. For a precise description of the role of the AHR-ARNT heterodimer, we generated an AHR mutant in which all amino acids described above were mutated (Fig. 1c).

**Figure 2:**
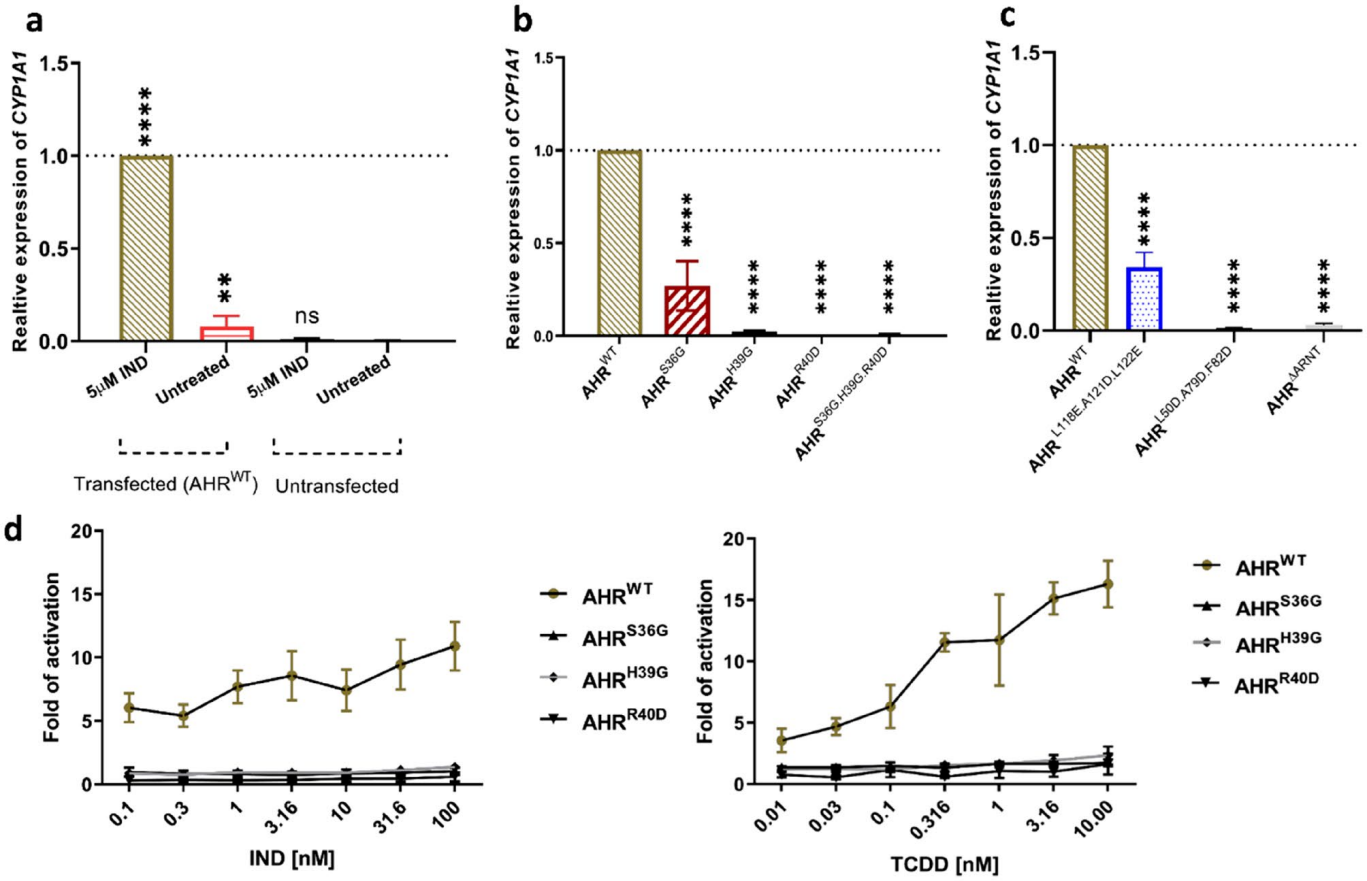
Transcriptional activity of the AHR and AHR mutants. Relative *CYP1A1* mRNA level determined by qPCR in MCF-7^ΔAHR^ cells. Values were standardized against *HPRT* and normalized against AHR^WT^ transfected cells treated with 5 µM indirubin (IND) for three hours. Depicted are the results for a comparison of treatment and AHR^WT^ transfection (**a**), AHR DNA-binding deficient mutants (**b**) and AHR mutants deficient for ARNT dimerization (**c**). (**d**) Luciferase activity driven by an XRE-promoter after stimulation with increasing concentrations of IND and TCDD for twenty-four hours. (**a**–**c**) Each bar represents the mean of three biological replicates ± S.D., One way Anova (ns: not significant, ** P < 0.01, **** P < 0.0001). (**d**) Presented are the means of three biological replicates + /- S.D.

In order to verify the generated AHR mutants, we analyzed their transcriptional activity as functional output in MCF-7^ΔAHR^ cells. *CYP1A1* mRNA levels were determined after induction of the AHR or the AHR mutants with IND, respectively (Fig. 2a–c). A mutation in the DNA-binding motif leads to a loss of transcriptional activity of the AHR. While AHR^H39G^ and AHR^R40D^ inhibit *CYP1A1* induction completely, AHR^S36G^ still shows a minor induction (Fig. 2b). Further analysis examined the XRE driven luciferase activity after treating MCF-7^ΔAHR^ cells with an increased concentration of TCDD or IND. In contrast to wild-type AHR, which activates luciferase in a concentration-dependent manner, the AHR DNA-binding deficient mutants do not elicit any reaction to increased concentrations of the ligands (Fig. 2d).

The transcriptional activity of ARNT-binding deficient mutants confirms that mutations in the bHLH domain (L50D.A79D.F82D) are sufficient to inhibit the induction of *CYP1A1* (Fig. 2c)*.* Nevertheless, the induction of *CYP1A1* is not inhibited completely through the mutation in the PAS-A domain (L118E.A121D.L122E) (Fig. 2c). Based on these results, we decided to solely consider the mutation L50D.A79D.F82D.L118E.A121D.L122E (AHR^∆ARNT^), which covers the ARNT-binding motif in both domains. Furthermore, a loss of the ARNT-dimerization ability of AHR^∆ARNT^ has been validated with co-immunoprecipitation (Co-IP) (Supplementary Fig. 1).

### The DNA-binding motif alters the nucleo-cytoplasmic distribution of AHR through the overlap with the second NLS

To investigate the cellular compartmentalization of the AHR in living HepG2 cells, human AHR was expressed as a fusion protein, linked to the enhanced yellow fluorescent protein (EYFP) as described previously (Tkachenko et al., 2016). The EYFP-tagged AHR or AHR mutants allowed us to perform online fluorescence imaging in living cells.

**Figure 3:**
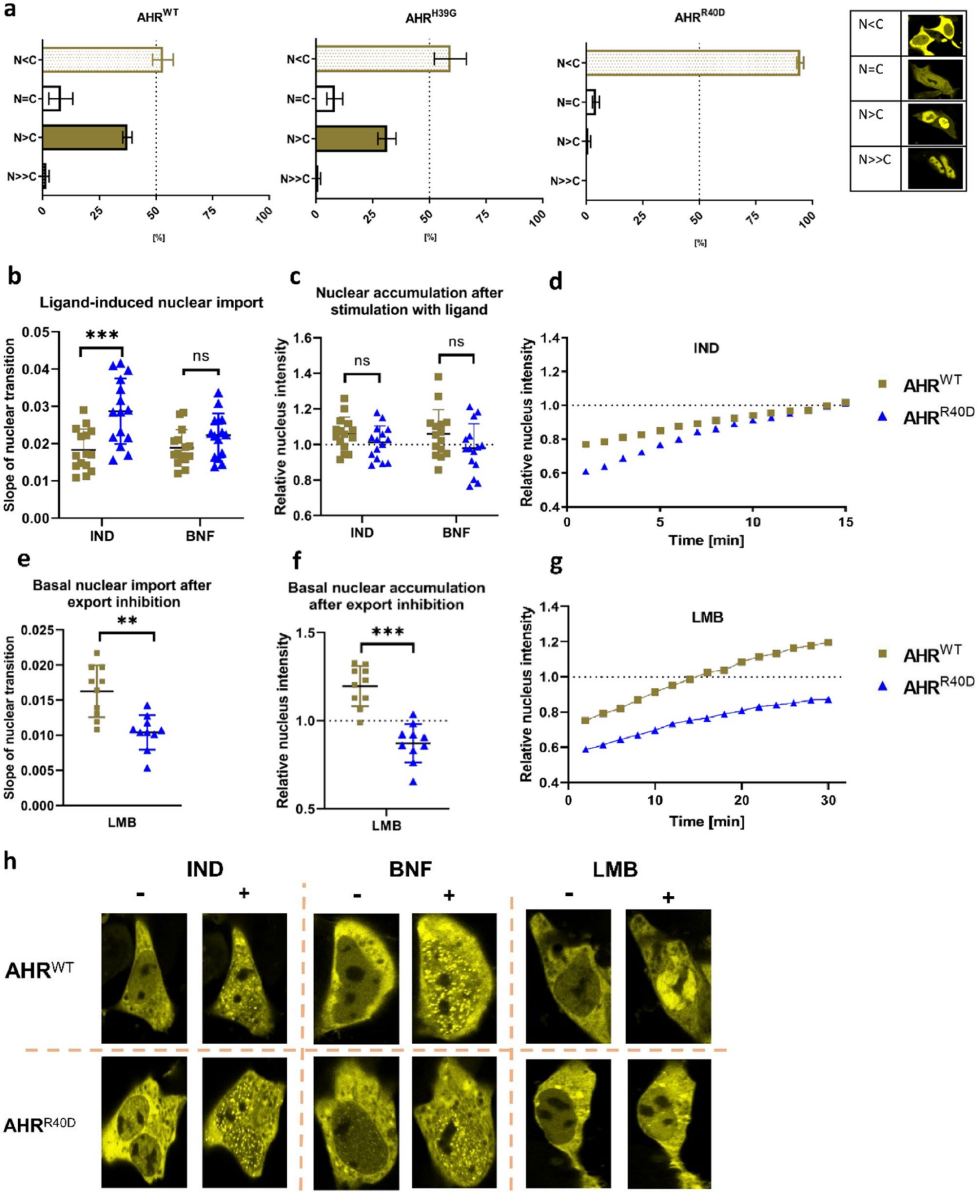
The DNA-binding motif determines the intracellular distribution of the AHR through the overlap with the second nuclear localization signal (NLS). (**a**) Distribution of the EYFP-tagged AHR and AHR mutants in HepG2 cells are shown. More than 300 cells were randomly selected and classified according to the given distribution pattern. Data represents mean ± S.D from three biological replicates. (**b**,**e**) Slopes of nuclear transition the AHR^WT^ and AHR^R40D^ (Arg → Asp) after treatment with 10 µM indirubin (IND), 10 µM β-naphthoflavone (BNF) or 200 nM leptomycin B (LMB). (**c**,**f**) Nuclear accumulation of the AHR^WT^ and AHR^R40D^ 15 min or 30 min after treatment with IND, BNF or LMB, respectively. Individual values and the mean ± S.D. of 15 (**b**,**c**) or 10 (**e**,**f**) cells are presented (two way ANOVA, Dunnett's multiple comparisons test, *** p < 0.001). (**d**,**g**) Representative measurements of time-lapse experiments after stimulation with IND (**d**) or LMB (**g**) of indicated AHR variants in HepG2 cells. Relative nuclear intensity denotes the intensity of the nucleus against the intensity of the entire cell. (**h**) Representative images of HepG2 cells transfected with AHR^WT^ or AHR^R40D^ before and after 15 min of treatment with IND, BNF, and LMB.

The DNA-binding motif includes two positive charged amino acids H39 and R40 that are considered to be a part of the 2nd NLS (Fig. 1a). Changing the weakly alkaline H39 to the neutral glycine (AHR^H39G^) did lead to a compartmentalization similar to the wild-type receptor (Fig. 3a). In contrast to AHR^H39G^, the distribution of the AHR^R40D^ mutant was shifted to an exclusively cytoplasmic distribution (Fig. 3a), suggesting that the basal nuclear import was abolished due to inactivation of the second NLS motif. From a technical perspective, AHR^H39G^ was confirmed as AHR mutant that is selectively deficient for DNA-binding, but contains a fully functional NLS.

On the other hand, AHR^R40G^ allows to address specifically the impact of the 2nd NLS on nuclear import. Interestingly, in response to the endogenous ligand (IND), nuclear accumulation was not delayed, but even accelerated when compared to wild-type AHR (Fig. 3b–d). Consistently, the xenobiotic ligand (BNF) did induce similar import profiles for AHR^R40D^ and wild-type receptor (Fig. 3b,c,h). These data suggest that the 2nd NLS has no apparent function in the ligand induced nuclear import.

To verify this hypothesis, we compared the basal import of AHR^R40D^ with the wild-type after blocking the protein export with leptomycin B (LMB). The subcellular distribution of AHR was observed until 30 min after treatment. Our results reveal that both the nuclear accumulation and the slope of nuclear transition of AHR^R40D^ is significantly reduced when compared with the wild-type (Fig. 3e–h), thus confirming a function of the 2nd NLS in the basal, but not in a ligand-induced nuclear import.

### Activation related nuclear retention of the AHR neither requires dimerization with ARNT nor binding to XRE

AHR heterodimerization with ARNT is the key to form a stable complex with the XRE motif. As ARNT is a nuclear protein that mediates AHR activation, we decided to investigate the requirement of the ARNT-AHR heterodimer for AHR nucleo-cytoplasmic translocation. The AHR mutant AHR^H39G^, containing a mutation within the DNA-binding domain, failed to activate *CYP1A1* expression (Fig. 2b). We used the mutants AHR^H39G.∆ARNT^and AHR^H39G^ to test, whether DNA-binding of the AHR or the formation of the corresponding transcription complex is a prerequisite for the maintenance of stable nuclear localization.

**Figure 4:**
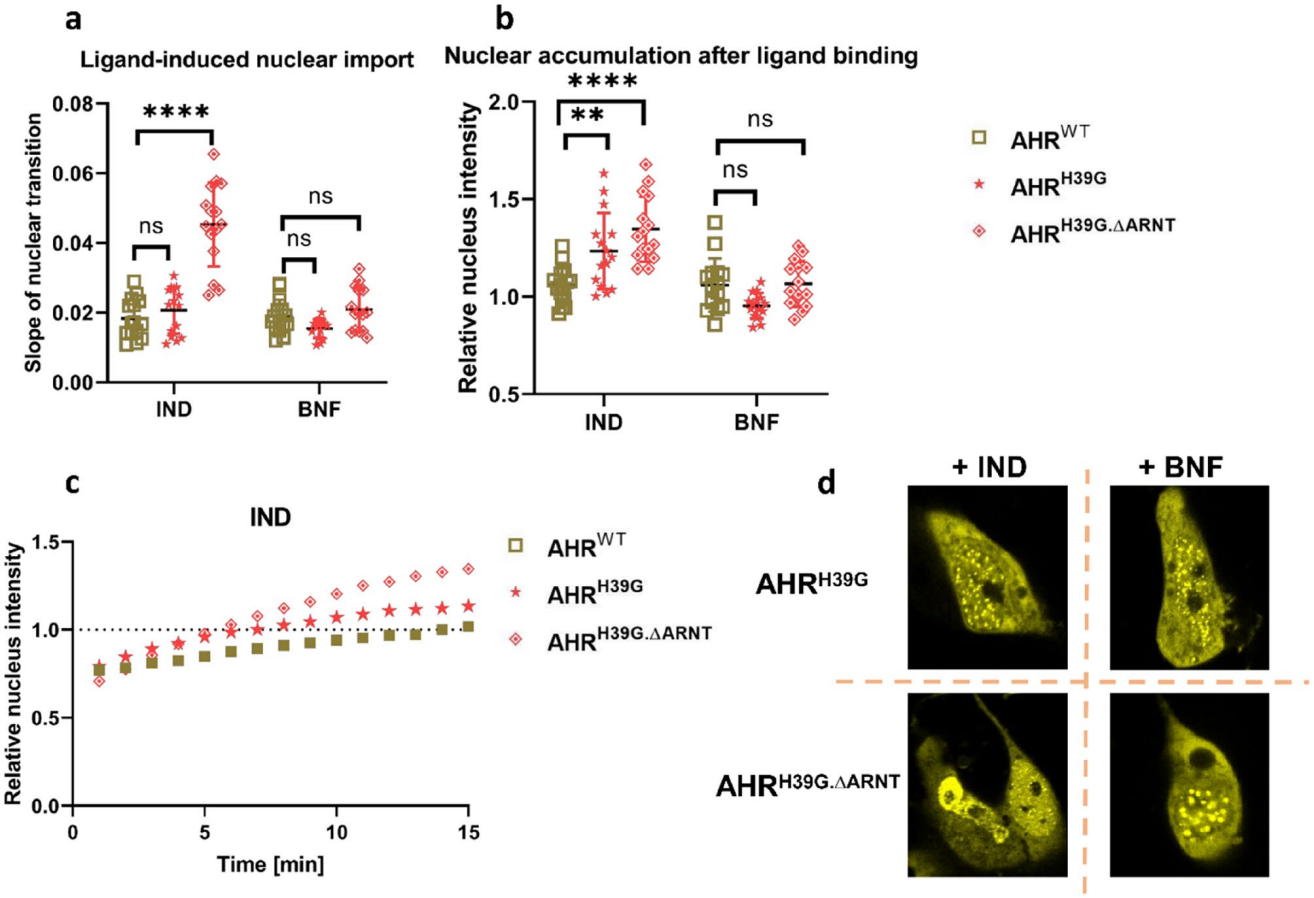
Forming the AHR-ARNT heterodimer and the active binding to the XRE is not decisive for stable ligand-dependent nuclear retention. (**a**) Slopes of nuclear transition of the AHR^WT^, AHR^H39G^ and AHR^H39G.∆ARNT^ after treatment with10µM indirubin (IND) or 10 µM β-naphthoflavone (BNF). (**b**) Nuclear accumulation of the AHR^WT^, AHR^H39G^ and AHR^H39G.∆ARNT^ 15 min after treatment with IND or BNF, respectively. Data display the individual values and the mean + /- S.D. of 15 cells (two way ANOVA, Dunnett's multiple comparisons test, ** p < 0.01, *** p < 0.001). (**c**) Representative measurements of time-lapse experiments after stimulation with IND of indicated AHR variants in HepG2 cells. Relative nucleus intensity denotes the intensity of the nucleus against the intensity of the entire cell. (**d**) Snapshots of HepG2 cells 15 min after stimulating with IND or BNF.

Analyzing intracellular trafficking of the AHR mutants after treatment with IND or BNF showed that both mutants AHR^H39G^ and AHR^H39G.∆ARNT^ exhibited a stronger nuclear accumulation after stimulation with the endogenous IND, as compared with the xenobiotic ligand BNF (Fig. 4b–d). Furthermore, the mutant AHR^H39G.∆ARNT^ shows significantly accelerated import after stimulation with IND (Fig. 4a).

This data indicates that the formation of the AHR-ARNT heterodimer and subsequent binding to the XRE is not crucial to stabilize the ligand-dependent nuclear retention of the AHR.

### Transient knockdown of ARNT does not change the AHR nucleo-cytoplasmic profile in MCF-7 cells

**Figure 5:**
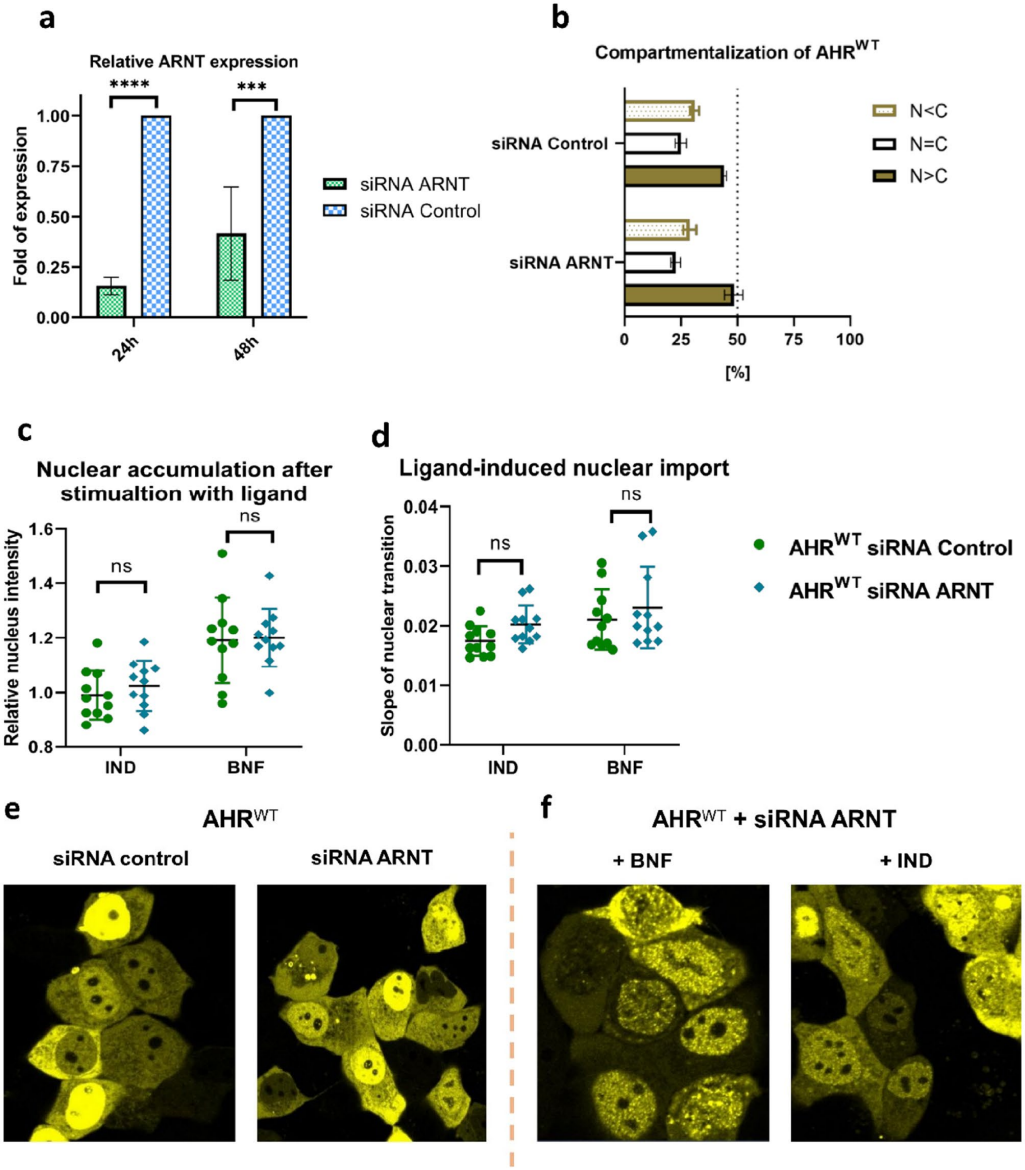
Down-regulation of ARNT is not critical for the AHR nucleo-cytoplasmic translocation in MCF-7 cells. (**a**) Relative *ARNT* mRNA level determined by qPCR in MCF-7^ΔAHR^ cells after transfection with siRNA against ARNT and control siRNA, respectively. Values were standardized against *HPRT* and normalized against control siRNA transfected cells. Each bar depicts the mean of three biological replicates ± S.D. (two way ANOVA, Dunnett's multiple comparisons test, *** p < 0.001,**** P < 0.0001). (**b**) Intracellular distribution pattern of EYFP-tagged AHR^WT^ in MCF-7 ^ΔAHR^ cells are shown. At least 300 transfected cells are randomly selected and sorted according to the following classification: N > C predominantly nuclear; N = C equal distribution; N < C predominantly cytoplasmic. Each bar represents the mean + /- S.D. of three biological experiments. Depicted are relative nuclear intensity (**c**) and slope (**d**) of time-lapse measurements after stimulation with 10 µM indirubin (IND) or 10 µM β-naphthoflavone (BNF) in control siRNA and ARNT siRNA transfected MCF-7^ΔAHR^ cells. The presented data contains individual values and the mean + /- S.D. of 11 cells. (**e**) Snapshots of MCF-7 ^ΔAHR^ cells before and after knocking down the ARNT gene. (**f**) Snapshots of the MCF-7 cells after stimulation with BNF and IND.

MCF-7 ^**ΔAHR**^ cells show a significant decrease in *ARNT* mRNA at 24 and 48 h after transfection with siRNA against *ARNT* (Fig. 5a). In the basal state, AHR^WT^ is predominantly located in the nucleus and knockdown of ARNT does not change the intracellular distribution (Fig. 5b,e). Interestingly, neither the ligand dependent nuclear accumulation nor the nuclear import after stimulation with IND or BNF are changed through ARNT knockdown (Fig. 5c,d,f). In conclusion, live tracking of AHR^WT^ in ARNT knockdown cells clearly indicates that nuclear import and accumulation caused by ligand binding is independent from ARNT mediated interactions.

## Discussion

In the absence of exogenous ligands, the AHR shuttles continuously between the cytoplasm and the nucleus while a predominantly cytoplasmic compartmentalization is maintained due to an efficient nuclear export. This “shuttling” has been previously reported as a constitutive process that does not lead to any transcriptional activity. Ligand binding accelerates nuclear import and sequesters the receptor in the nucleus. Importantly, this is linked to an exit from the shuttling cycle and to a deactivated export, which determines the cytoplasmic localization prior to activation. However, the molecular mechanisms that stabilize the nuclear AHR population have not been characterized yet.

A prior study of our lab defined the Q-rich/PST domain in the C-terminus as a crucial component for regulating both the shuttling and the ligand-induced import^29^. In particular, the Q-rich/PST domain is essential for regulating intracellular trafficking and nuclear accumulation after ligand binding. The C-terminal AHR deletion mutant AHR^∆647^, lacking 201 residues after valine 647, did not show any nuclear accumulation in response to ligands due to increased export. Intriguingly, point mutations of valine 647 (AHR^V647A^), or C-terminal deletions that exclude this residue (AHR^∆646^ or shorter) display consistently an exclusive nuclear staining. These previous data implied a mandatory role of valine 647 to maintain a conformation that is capable to undergo export. Further, the adjacent C-terminal domain is required to switch off export during activation, possibly in concert with the well-studied N-terminal trafficking motifs.

In this manuscript, we have addressed the impact of the N-terminal domain on both shuttling and ligand-induced import. Besides the well characterized nuclear transport signals, this domain harbors the DNA-binding XRE motif and participates in the heterodimerization with ARNT that is regarded as crucial step towards activation. There are now two hypotheses for the molecular mechanism of the AHR activation cascade. Firstly, ligand binding might trigger a conformational change that accelerates import and blocks nuclear export in parallel. This would enable all consecutive interactions that lead to transcription of target genes and other nuclear effects. Alternatively, termination of export could be linked to molecular interactions of the AHR that are formed during activation. In order to distinguish both options, we have analyzed whether the DNA-binding motif or the interaction with ARNT are required to stabilize the nuclear AHR fraction during activation. In our experiments, we have used IND as an endogenous ligand, as well as BNF as a xenobiotic agonist. Further, we verified that all AHR mutants, deficient of DNA or ARNT-binding, did not induce transcription of *CYP1A1*.

As the DNA-binding motif overlaps with the 2nd NLS, mutagenesis might also affect nuclear import. Indeed, partial inactivation of nuclear import was indicated for AHR^R40D^ by an absence of basal nuclear staining. We have aimed to separate both functions, generating a mutant that is deficient to bind the XRE without compromising the second section of the bipartite NLS. AHR^H39G^ met the requirement of a comparable basal staining as the wild-type receptor and was used to address the impact of the DNA-binding domain on ligand-induced nuclear import. Importantly, the kinetics of nuclear accumulation were similar for both to the wild-type AHR and AHR^H39G^. Surprisingly, this was also true for AHR^R40D^, suggesting that neither DNA-binding nor the second section of the NLS affect the ligand-induced nuclear retention.

We further used the mutant AHR^R40D^ to characterize the 2nd NLS in more detail. Interestingly, this mutant failed to accumulate in the nucleus after protein export was inhibited using LMB. Our data indicate a specific role for this trafficking motif in the basal (i.e. ligand-independent) nuclear import that occurs in the context of nucleo-cytoplasmic shuttling. In contrast, this motif has no impact on the ligand induced nuclear transfer. These observations underscore the idea that the ligand induced and the basal import constitute two independent transport mechanisms. The basal import might take place due to a direct binding to importin-β through the arginine rich NLS. Findings by Pterulis et al. further support this hypothesis by showing that the murine AHR is able to perform a stable binding with importin-β^26^. In conclusion, our results propose that the NLS stays partially unmasked in the absence of exogenous ligands, thereby enabling binding with importin-β and allowing the basal nuclear import. However, this theory has to be addressed in a next study.

As second question, we have studied whether dimerization with ARNT is required to retain the AHR in the nucleus. The mutant AHR^H39G.∆ARNT^ is an inactive AHR variant with a similar compartmentalization as the wild type, that is incapable to dimerize with ARNT or bind the DNA. Data indicate that ligand-induced nuclear retention of the AHR does not depend on functional interactions with ARNT. This was also confirmed after inhibition of ARNT expression using siRNA. The transient knockdown of ARNT reveals that heterodimerization is not required for the AHR to maintain a stable nuclear localization.

Notably in comparison with wild type AHR, both AHR^H39G.∆ARNT^and AHR^H39G^ exhibit a strong increase in the nuclear accumulation after stimulation with the endogenous IND but not with the xenobiotic ligand BNF. For AHR^H39G.∆ARNT^ treated with IND, we also detected an increased slope of nuclear transition, which directly leads to higher nuclear accumulation. The reason for the change in nuclear transition, meaning nuclear import and parallel re-export in general, through IND opposed to BNF cannot be elucidated with our experiments. For AHR^H39G^, the nuclear transition was not significantly altered in comparison to wild type AHR and increase in nuclear accumulation was less pronounced. However, nuclear import and induction of target gene expression (*CYP1A1*) correlates for IND but less for BNF^4^. Based on this, one might hypothesize that nuclear transition and functional effectivity of AHR within the nucleus might be linked. Therefore, the effect is stronger for the more potent ligand IND than for BNF.

Taken together, our results indicate that the stabilized nuclear population of ligand-bound or ligand-free AHR is neither dependent on interactions with DNA nor the ARNT. Therefore, the kinetic of nuclear accumulation is not related to the molecular interactions conducted by the activated receptor in the nucleus. In conclusion to our data, we propose that the conformational change followed by ligand binding promotes the nuclear import and blocks the export autonomously.

## Methods

### Plasmids

The plasmid pEYFP-AHR-C1 used by^29^ encodes the human AHR (start at Ala 11). All pEYFP-AHR variants, which carry mutations at certain amino acids (AHR^H39G^, AHR^R40D^, AHR^S36G^, AHR^S36G.H39G.R40D^, AHR^L118E.A121D.L122E^, AHR^L50D.A79D.F82D^ and AHR^H39G.L50D.A79D.F82D.L118E.A121D.L122E.^) were generated by GenScript (GenScript Biotech, Leiden, Netherlands).

### Cell culture

The human hepatoma cell line HepG2 was purchased from DSMZ (Braunschweig, Germany). HepG2 cells were grown in RPMI 1640. MCF-7^∆AHR^ cells were cultured in DMEM. Both cell lines were maintained in 5% CO_2_ at 37 °C in culture medium containing 10% (v/v) FCS, 100 U/ml penicillin, 100 mg/ml streptomycin, and 2 mM L-glutamine. All media components were purchased from Pan-Biotech (Aidenbach, Germany). AHR-deficient variant of MCF-7 cells were kindly provided by Dr. P. Tarnow^37^.

### Transient transfection

HepG2 and MCF-7^∆AHR^ cells were seeded either on 10-cm dishes, 6-well plates (Techno Plastic Products AG, Trasadingen, Switzerland), 96-well plates (Techno Plastic Products AG, Trasadingen, Switzerland), or on glass bottom dishes (In VitroScientific, Sunyvale, CA, USA). On the next day, cells on glass bottom dishes were transfected by using Xfect (Takara Bio Europe SAS, Saint-Germain-en-Laye, France) and an appropriate DNA amount according to the manufacturer’s instructions. Cells on 10-cm dishes or on multiwell plates were transfected with Lipofectamine 2000 (Invitrogen, Carlsbad, CA, USA) and an appropriate DNA amount as stated by the manufacturer’s instructions. After 4 h incubation, transfection medium was removed and replaced by new RPMI or DMEM medium, respectively.

### Reagents

β-naphthoflavone (BNF) and dimethyl sulfoxide (DMSO) were obtained from Sigma-Aldrich (Sigma-Aldrich Chemie GmbH, Munich, Germany). Leptomycin B (LMB) was acquired from Santa Cruz Biotechnology (Santa Cruz Biotechnology Inc., Texas, U.S.A). Indirubin (IND) was provided from Enzo Life Sciences (Enzo Life Sciences GmbH, Lörrach, Germany) and 2,3,7,8-tetrachlordibenzo-*p*-dioxin (TCDD) from LGC Standards (LGC Standards GmbH, Wesel, Germany). All chemicals were purchased at the highest purity available.

### RNA analysis

RNA was isolated form cells by using RNeasy Midi kit in conjunction with QIAshredder (QIAGEN GmbH, Hilden, Germany). The purity and the concentration of RNA were determined using a plate reader device (Infinite M200 PRO, Tecan Trading AG, Männedorf, Switzerland). Reverse transcription was performed with the high-capacity cDNA Reverse Transcription kit (Applied Biosystems, Foster City, CA USA).

Quantitative PCR (qPCR) was performed on a 7500 Fast Real-Time PCR instrument using FAST SYBR Green Master Mix (Applied Biosystems, Foster City, CA, USA). Hypoxanthine–guanine phosphoribosyl transferase (*HPRT*) was used as reference gene.

The primer sequences are listed in Table 1.

**Table 1:** Primer sequences used.

Gene	Forward primer	Reverse primer	Product size (bp)
*CYP1A1*	5′- CCAAGAGTCCACCCTTCCCAGCT -3′	5′- GAGGCCAGAAGAAACTCCGTGGC -3′	371
*HPRT*	5′-GTTCTGTGGCCATCTGCTTAG-3′	5′-GCCCAAAGGGAACTGATAGTC-3′	144

### Luciferase assay

Cells used for the luciferase assay were seeded in 96-well plates (Merck KGaA, Darmstadt, Germany) at a concentration of 4.5 × 10^4^ and 150 µL per well. Cell were transfected with pGL4.26-XRE provided by Dr. P. Tarnow^38^ together with AHR plasmids (WT or mutants). 24 h after stimulation cells were washed with PBS and lysed with lysis-puffer (10 mM Tris 0.1 mM, 2 mM EDTA, 1% Triton™ X-100) for 15 min. 50 µl luciferin solution and 150 µl assay puffer (Gly-Gly puffer, DTT and ATP) were injected per well. Luminescence was measured in a Synergy Neo2 plate reader (BioTek, Bad Friedrichshall, Germany).

### RNA-silencing

For RNA silencing, cells were transfected with 80 pmol of siRNA (sc-29734) or the control siRNA (sc-37007) (all from Santa Cruz Biotechnology, Heidelberg, Germany) by using Lipofectamine 2000 according to the manufacturer’s instructions. The expression of the *ARNT* gene was measured by using the ARNT primer (sc-29734-PR) provided from Santa Cruz Biotechnology.

### On-line confocal microscopy

HepG2 and MCF-7^∆AHR^ cells are seeded and transfected as described above. Cells were monitored by confocal microscope (LSM 700, Carl Zeiss Jena GmbH, Jena, Germany) twenty four hours post transfection. For live cell imaging, cells were maintained in buffered medium in 5% CO_2_ at 37 °C. Simultaneously with the treatments, the imagining was initiated at a rate of one picture per minute. Microscopic image acquisition was done by using ZEN 2012 black edition software (Carl Zeiss Jena GmbH). For data analysis ZEN 2012 blue edition was used (Carl Zeiss Jena GmbH). The nucleus and the whole cell were defined and outlined as a region of interest (ROI), for which the fluorescence intensity was extracted.

### Statistics

Data were analysed and graphed using GraphPad Prism (Graph Pad, La Jolla, CA, USA). Statistical analysis was done using paired Dunnett's multiple comparisons test, one-way or two-way ANOVA, * p < 0.01, ** p < 0.01, *** p < 0.001, **** p < 0.0001.

## Supplementary Information

Supplementary Information.

## References

[1] Beischlag TV (2008). The aryl hydrocarbon receptor complex and the control of gene expression. Crit. Rev. Eukaryot. Gene Exp..

[2] Chen G, Bunce NJ (2004). Interaction between halogenated aromatic compounds in the Ah receptor signal transduction pathway. Environ. Toxicol..

[3] Luch A (2005). Nature and nurture—lessons from chemical carcinogenesis. Nat. Rev. Cancer.

[4] Tkachenko A (2018). Nuclear transport of the human aryl hydrocarbon receptor and subsequent gene induction relies on its residue histidine 291. Arch. Toxicol..

[5] Puga A, Ma C, Marlowe JL (2009). The aryl hydrocarbon receptor cross-talks with multiple signal transduction pathways. Biochem. Pharmacol..

[6] Tarnow T, Luch A (2019). Chemical activation of estrogen and aryl hydrocarbon receptor signaling pathways and their interaction in toxicology and metabolism. Exp. Opin. Drug Metab. Toxicol..

[7] Carreira VS (2015). Ah receptor signaling controls the expression of cardiac development and homeostasis genes. Toxicol. Sci..

[8] Walisser JA, Glover E, Pande K, Liss AL, Bradfield CA (2005). Aryl hydrocarbon receptor-dependent liver development and hepatotoxicity are mediated by different cell types. Proc. Natl. Acad. Sci. U.S.A..

[9] Neavin DR, Liu D, Ray B, Weinshilboum RM (2018). The role of the aryl hydrocarbon receptor (AHR) in immune and inflammatory diseases. Int. J. Mol. Sci..

[10] Guerrina N, Traboulsi H, Eidelman DH, Baglole CJ (2018). The aryl hydrocarbon receptor and the maintenance of lung health. Int. J. Mol. Sci..

[11] van den Bogaard EH (2015). Genetic and pharmacological analysis identifies a physiological role for the AHR in epidermal differentiation. J. Invest. Dermatol..

[12] Rannug A (2020). How the AHR became important in intestinal homeostasis-a diurnal FICZ/AHR/CYP1A1 feedback controls both immunity and immunopathology. Int. J. Mol. Sci..

[13] Zelante T (2013). Tryptophan catabolites from microbiota engage aryl hydrocarbon receptor and balance mucosal reactivity via interleukin-22. Immunity.

[14] Nguyen LP, Bradfield CA (2008). The search for endogenous activators of the aryl hydrocarbon receptor. Chem. Res. Toxicol..

[15] Adachi J (2001). Indirubin and indigo are potent aryl hydrocarbon receptor ligands present in human urine. J. Biol. Chem..

[16] Moura-Alves P (2014). AhR sensing of bacterial pigments regulates antibacterial defence. Nature.

[17] Murray IA, Patterson AD, Perdew GH (2014). Aryl hydrocarbon receptor ligands in cancer: Friend and foe. Nat. Rev. Cancer.

[18] Wu D, Potluri N, Lu J, Kim Y, Rastinejad F (2015). Structural integration in hypoxia-inducible factors. Nature.

[19] Kolonko M, Greb-Markiewicz B (2019). bHLH-PAS proteins: Their structure and intrinsic disorder. Int. J. Mol. Sci..

[20] Brunnberg S (2003). The basic helix-loop-helix-PAS protein ARNT functions as a potent coactivator of estrogen receptor-dependent transcription. Proc. Natl. Acad. Sci. U.S.A..

[21] Rüegg J (2008). The transcription factor aryl hydrocarbon receptor nuclear translocator functions as an estrogen receptor beta-selective coactivator, and its recruitment to alternative pathways mediates antiestrogenic effects of dioxin. Mol. Endocrinol..

[22] Ikuta T, Eguchi H, Tachibana T, Yoneda Y, Kawajiri K (1998). Nuclear localization and export signals of the human aryl hydrocarbon receptor. J. Biol. Chem..

[23] Ikuta T (2000). Nucleocytoplasmic shuttling of the aryl hydrocarbon receptor. J. Biochem..

[24] Lange A (2007). Classical nuclear localization signals: Definition, function, and interaction with importin alpha. J. Biol. Chem..

[25] Rowlands JC, Gustafsson JA (1997). Aryl hydrocarbon receptor-mediated signal transduction. Crit. Rev. Toxicol..

[26] Petrulis JR, Kusnadi A, Ramadoss P, Hollingshead B, Perdew GH (2003). The hsp90 Co-chaperone XAP2 alters importin beta recognition of the bipartite nuclear localization signal of the Ah receptor and represses transcriptional activity. J. Biol. Chem..

[27] Murray IA (2005). Evidence that ligand binding is a key determinant of Ah receptor-mediated transcriptional activity. Arch. Biochem. Biophys..

[28] Moroianu J (1999). Nuclear import and export pathways. J. Cell. Biochem..

[29] Tkachenko A (2016). The Q-rich/PST domain of the AHR regulates both ligand-induced nuclear transport and nucleocytoplasmic shuttling. Sci. Rep..

[30] Tsuji N (2014). The activation mechanism of the aryl hydrocarbon receptor (AhR) by molecular chaperone HSP90. FEBS Open Bio.

[31] Swanson HI, Chan WK, Bradfield CA (1995). DNA binding specificities and pairing rules of the Ah receptor, ARNT, and SIM proteins. J. Biol. Chem..

[32] Hestermann EV, Brown M (2003). Agonist and chemopreventative ligands induce differential transcriptional cofactor recruitment by aryl hydrocarbon receptor. Mol. Cell. Biol..

[33] Davarinos NA, Pollenz RS (1999). Aryl hydrocarbon receptor imported into the nucleus following ligand binding is rapidly degraded via the cytosplasmic proteasome following nuclear export. J. Biol. Chem..

[34] Schulte KW, Green E, Wilz A, Platten M, Daumke O (2017). Structural basis for aryl hydrocarbon receptor-mediated gene activation. Structure.

[35] Seok SH (2017). Structural hierarchy controlling dimerization and target DNA recognition in the AHR transcriptional complex. Proc. Natl. Acad. Sci. U.S.A..

[36] Wu D, Potluri N, Kim Y, Rastinejad F (2013). Structure and dimerization properties of the aryl hydrocarbon receptor PAS-A domain. Mol. Cell. Biol..

[37] Tarnow P (2020). Characterization of quinoline yellow dyes as transient aryl hydrocarbon receptor agonists. Chem. Res. Toxicol..

[38] Tarnow P (2017). A novel dual-color luciferase reporter assay for simultaneous detection of estrogen and aryl hydrocarbon receptor activation. Chem. Res. Toxicol..

