# Supplementary material for: The role of DNA-binding and ARNT dimerization on the nucleo-cytoplasmic translocation of the aryl hydrocarbon receptor
Source: Sci Rep. 2021 Sep 14;11:18194. doi: 10.1038/s41598-021-97507-w (PMC8440571; doi:10.1038/s41598-021-97507-w)
Supplement: Supplementary file 1 — Supplementary Information. [file 41598_2021_97507_MOESM1_ESM.pdf]

Supplementary information to:

The Role of DNA-Binding and ARNT Dimerization on the Nucleo-Cytoplasmic Translocation of the Aryl Hydrocarbon Receptor

Rashad Haidar[1-2]*, Frank Henkler[1], Josephine Kugler[1], Aline Rosin[1], Doris Genkinger[1], Peter Laux[1], Andreas Luch[1-2]

[1] German Federal Institute for Risk Assessment (BfR), Department of Chemical and Product Safety, Berlin, Germany

[2] Institute of Pharmacy, Department of Biology, Chemistry and Pharmacy, Freie Universität Berlin, Berlin, Germany

* Correspondence should be addressed to Rashad.Haidar@bfr.bund.de

Supplementary materials and methods

Co-Immunoprecipitation. MCF-7$^{\Delta AHR}$ cells were seeded on 10-cm dishes (Techno Plastic Products AG, Trasadingen, Switzerland). On the next day, cells were transfected by using 24 µg pEYFP-AHR (WT or mutant) and 16 µg pmCherry-ARNT with 40 µl Lipofectamine 2000 (Invitrogen, Carlsbad, CA, USA) according to the manufacturer's instructions. For the negative control, cells were transfected with pmCherry-ARNT alone. After 4 hours incubation, transfection medium was removed and replaced by new cultivation medium. After 24 hours, cells were treated with 5 µM Indirubin for three hours. For co-immunoprecipitation the GFP-Trap® Magnetic Agarose (Chromotek, Planegg, Germany) was used according to the manufacturer's instructions. Cell extracts were incubated with GFP-trap beads for two hours at 4°C. For each sample, 15 µg supernatant was saved as input for Western blots. YFP protein were eluted with SDS-sample buffer and then tested by western blotting.

Western-blot. Cells were lysed on ice in RIPA buffer (10 mM Tris/Cl pH 7.5, 150 mM NaCl, 0.5 mM EDTA, 0.1 % SDS, 1 % Triton™ X-100, 1 % deoxycholate) with protease inhibitor cocktail (Calbiochem, San Diego, CA, USA) and DNase (New England Biolabs, Ipswich; England). Protein concentration was measured with the Pierce™ BCA Protein Assay Kit (Thermo Scientifc, Waltham, MA, USA). Equal amounts of proteins were applied to SDS-PAGE, transferred onto nitrocellulose membranes and immunoblotted according to the manufacturer's instructions. The primary antibodies were, anti-AHR used at 1:300 (sc-133088) and anti-ARNT used at 1:600 (sc-17811) (all from Santa Cruz Biotechnology, Heidelberg, Germany). Primary antibodies were labelled with appropriate horseradish peroxidase (HRP)-coupled secondary antibodies (Santa Cruz Biotechnology) and visualized with Pierce ECL Substrate (Thermo Fisher Scientifc, Waltham, MA, USA). As a loading control, HRP conjugated anti-beta Actin was used at 1:12,500 (ab 49900, Abcam, Cambridge, UK).

Supplementary Figures

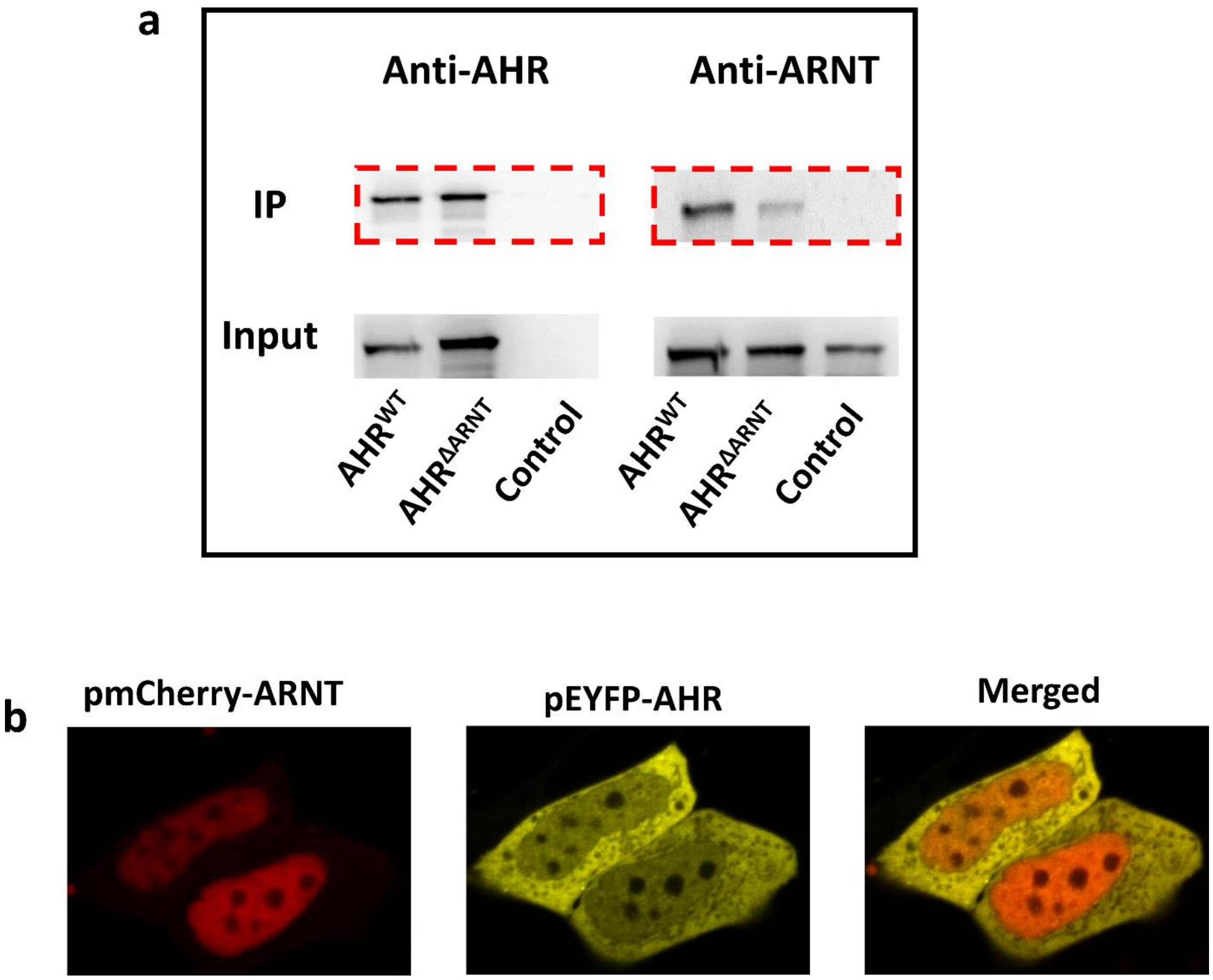

Suppl Fig 1. Co-Immunoprecipitation: (a) Cropped Western Blot images after co-immunoprecipitation of input (lower lane) and precipitate (upper row) for AHR (left) and ARNT (right) for indicated constructs and pmCherry-ARNT. Full-length blots are presented in Suppl Fig 2 **(b)** Representative image of MCF-7$^{\Delta AHR}$ cells co-transfected with pEYFP-AHR (yellow) and pmCherry-ARNT (red).

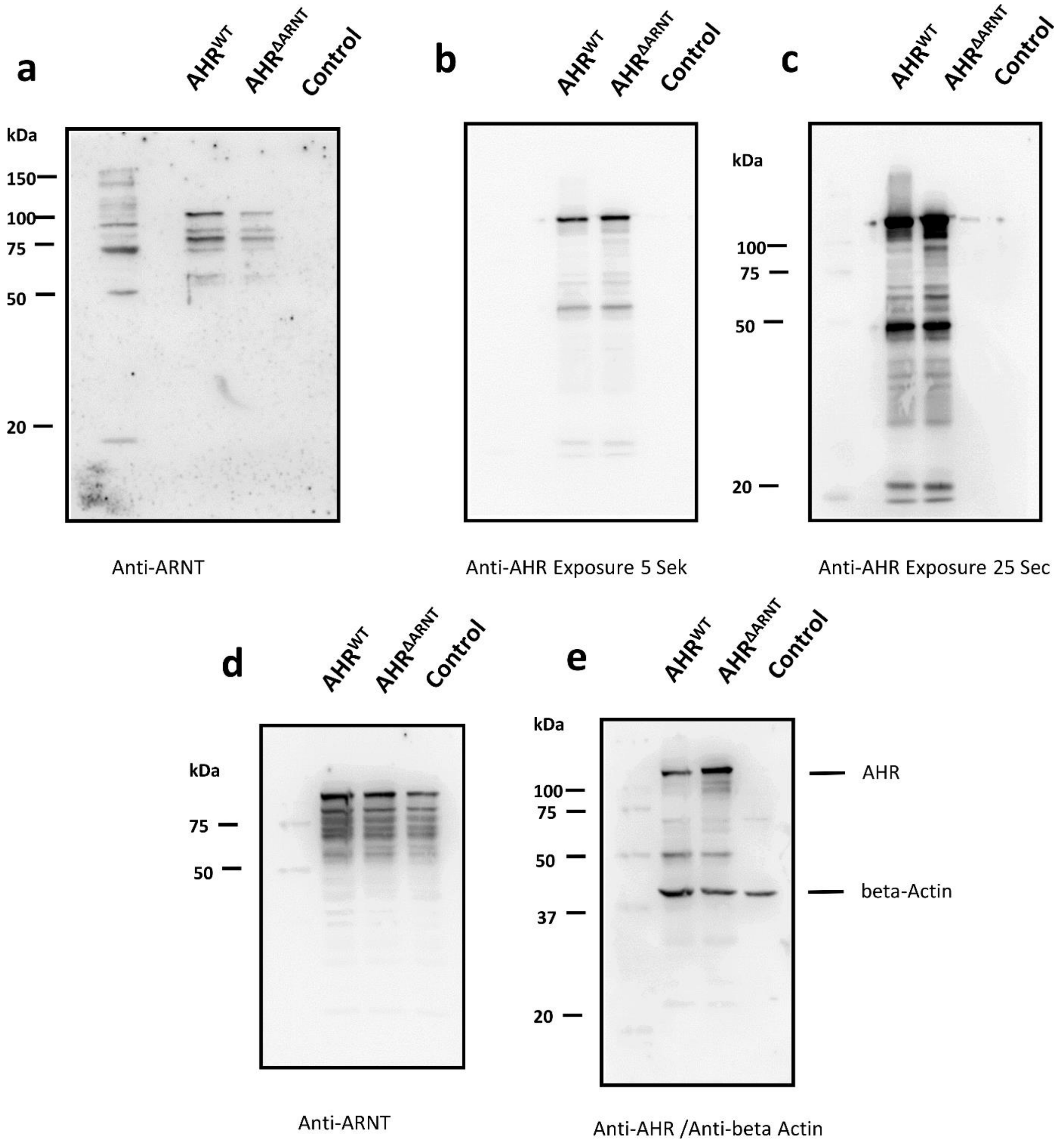

Suppl Fig 2.Full length blots of the cropped Western Blot images from Suppl Fig 1 after co-immunoprecipitation of precipitate for ARNT **(a)**, AHR after 5 / 25 seconds exposure **(b)/(c)** and input for ARNT **(d)**, AHR and beta Actin **(e)**.
